# Advances in metabolic reprogramming of NK cells in the tumor microenvironment on the impact of NK therapy

**DOI:** 10.1186/s12967-024-05033-w

**Published:** 2024-03-03

**Authors:** Linxuan Miao, Chenglin Lu, Bin Zhang, Huili Li, Xu Zhao, Haoran Chen, Ying Liu, Xiaonan Cui

**Affiliations:** 1 Department of Oncology, The First Affiliated Hospital of Dalian Medical University, Dalian, 116011 People’s Republic of China; 2 Institute (College) of Integrative Medicine, Dalian Medical University, Dalian, 116000 People’s Republic of China; 3 Department of Oncology, Affiliated Zhongshan Hospital of Dalian University, Dalian, 116001 People’s Republic of China

**Keywords:** Tumor microenvironment, Natural killer cells, Immunotherapy, Solid tumors

## Abstract

Natural killer (NK) cells are unique from other immune cells in that they can rapidly kill multiple neighboring cells without the need for antigenic pre-sensitization once the cells display surface markers associated with oncogenic transformation. Given the dynamic role of NK cells in tumor surveillance, NK cell-based immunotherapy is rapidly becoming a "new force" in tumor immunotherapy. However, challenges remain in the use of NK cell immunotherapy in the treatment of solid tumors. Many metabolic features of the tumor microenvironment (TME) of solid tumors, including oxygen and nutrient (e.g., glucose, amino acids) deprivation, accumulation of specific metabolites (e.g., lactate, adenosine), and limited availability of signaling molecules that allow for metabolic reorganization, multifactorial shaping of the immune-suppressing TME impairs tumor-infiltrating NK cell function. This becomes a key barrier limiting the success of NK cell immunotherapy in solid tumors. Restoration of endogenous NK cells in the TME or overt transfer of functionally improved NK cells holds great promise in cancer therapy. In this paper, we summarize the metabolic biology of NK cells, discuss the effects of TME on NK cell metabolism and effector functions, and review emerging strategies for targeting metabolism-improved NK cell immunotherapy in the TME to circumvent these barriers to achieve superior efficacy of NK cell immunotherapy.

## Introduction

NK cells are cytotoxic lymphocytes of the innate immune system, accounting for approximately 15% of all circulating lymphocytes, and their activation is driven by a balance between activating and inhibitory signals, therefore, they do not need to be sensitized beforehand to lysate target cells and thus exert antitumor effects [1]. Activated NK cells induce metabolic changes and drive effector functions including the release of cytolytic particles containing perforin and granzymes or the killing of infected or transformed cells by ligating death-inducing receptors (TRAIL, FasL). They also contribute to the development of adaptive immune responses by producing various chemokines and pro-inflammatory cytokines [2, 3]. NK cells are therefore a valuable therapeutic tool in cancer immunotherapy and a variety of NK cell-based immunotherapies have been developed, some of which have been translationally applied in the clinic [4, 5]. Encouraging efficacy has been shown in hematologic malignancies (e.g., myeloid leukemia, chronic lymphocytic leukemia, and acute lymphoblastic leukemia), but efficacy in the treatment of solid tumors is usually poor [6]. One of the main limiting factors is immunosuppressive TME. The function, phenotype, activation, and persistence of tumor-infiltrating NK cells are impaired in nutrient-depleted, hypoxic, acidic TME, even leading to NK cell dysfunction or exhaustion. Consider that cellular metabolism provides the energy and biosynthetic requirements of cells to support their effector functions. We focused on the effects of inhibitory TME on energy expenditure and metabolic reprogramming of NK cells, where understanding and regulating NK cell metabolism is critical to optimize the efficacy of current and future NK cell immunotherapies. Here, we summarize the metabolic biology of NK cells, discuss the effects of TME on NK cell metabolism and effector functions, and review emerging strategies for targeting metabolic improvement of NK cell immunotherapy in the TME.

## Immune metabolism of NK cells

### Biological characteristics of NK cell metabolism

**Fig. 1:**
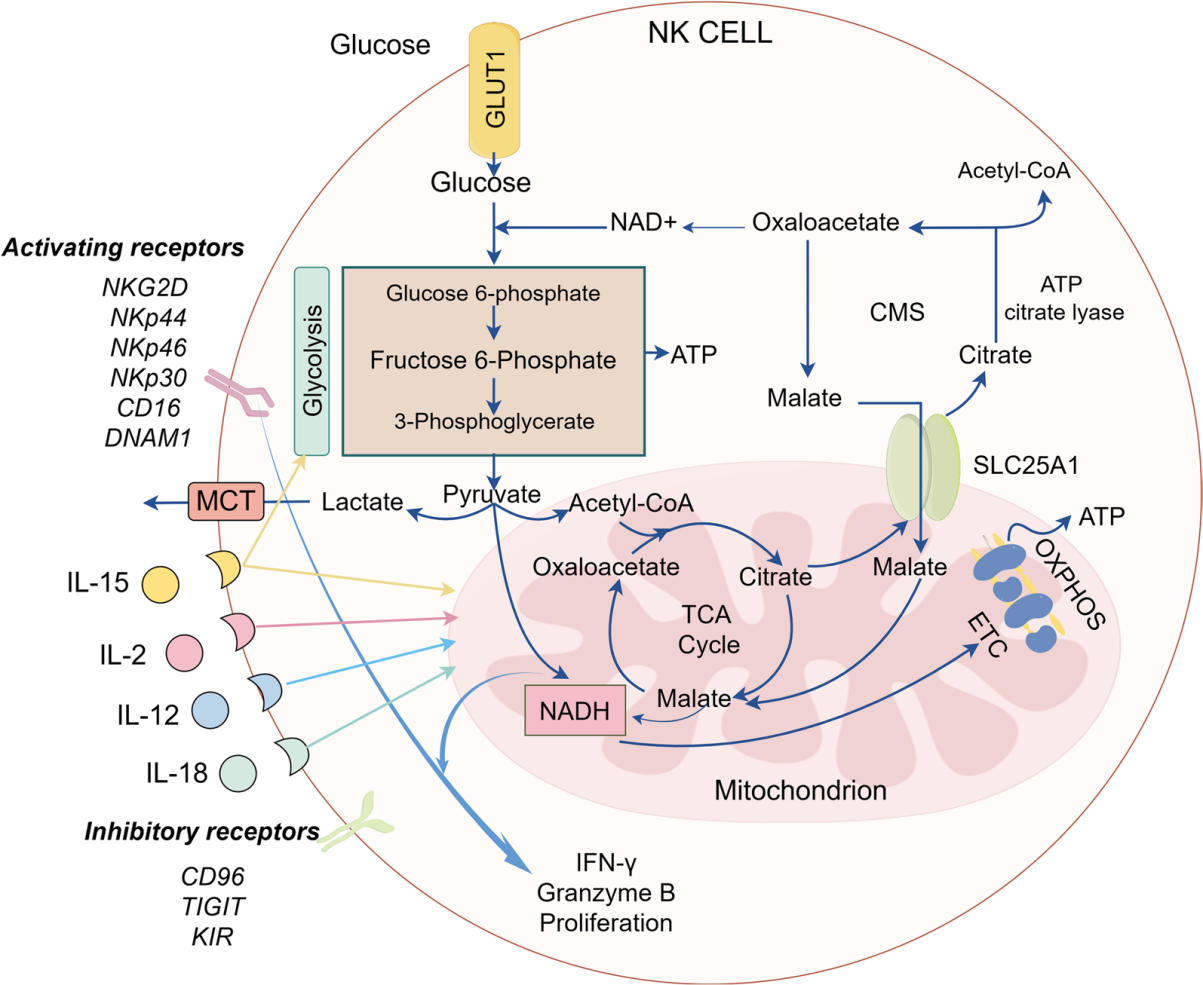
Citrate-Malate Shuttle (drawed by Figdraw)

Cell destiny can be determined by metabolic flux. Different developmental stages of NK cells exhibit distinct metabolic fluxes. Developing mouse NK cells undergo several stages of maturation in the bone marrow and can be recognized based on CD11b and CD27 expression levels. They are mainly categorized into immature stage CD11blowCD27hi, CD11blowCD27hi NK cells, intermediate stage CD11b hiCD27 hi NK cells and final developmental maturation stage CD11bhiCD27low NK cells [7]. NK cells' metabolic activity varies dynamically during development, and as they continue to differentiate and mature, they become less dependent on glycolysis and cellular glucose uptake [8]. Whereas immature NK cells express the amino acid transporter CD98 and the transferrin receptor CD71, which gives them increased metabolic activity to sustain their proliferation [8]. It has been discovered that mTOR, a metabolic regulator, plays a crucial role in the integration of NK cell growth and metabolism. In mTOR-deficient mice, NK cell development is halted in the bone marrow at the CD11bhiCD27hi stage, and NK cells are almost nonexistent in peripheral organs [8]. Mature, differentiated CD11bhiCD27low NK cells arrest and enter a resting state. Resting mouse and human NK cells have similar low baseline metabolic rates, maintaining low levels of glycolysis and oxidative phosphorylation (OXPHOS), according to several metabolic profile investigations on the cells [9–11]. Resting mouse NK cells have a low basal metabolic rate and maintain low levels of glycolysis and OXPHOS, and the metabolic activity of NK cells does not increase significantly under short bursts of cytokine stimulation or receptor signaling. However, this low metabolic rate is important for maintaining an acute NK cell response, and inhibition of OXPHOS or glycolysis would result in almost complete elimination of interferon γ (IFN-γ) produced by receptor stimulation [12]. Elevated OXPHOS levels are seen in human NK cells activated with short-term IL-12/15 or IL-2 cytokines; these levels are crucial for effective ATP production, which is needed to activate human NK cell role [11]. Both human and murine NK cells respond to prolonged and sustained cytokine stimulation by strong metabolic changes that meet the energy requirements for NK cells to carry out their effector functions. These changes include increases in mitochondrial mass, glycolytic enzyme expression, key nutrient transport proteins (e.g., SLC2A1 and SLC1A5), and glycolysis rates and OXPHOS ratios [9, 11, 13]. The timescales of metabolic reprogramming in human and mouse lymphocytes, however, may differ, according to recent research, with human lymphocytes demonstrating longer metabolic reprogramming times—this is proven for T lymphocytes, but more evidence is required for NK cells [14]. The aforementioned results direct our emphasis toward the topic of how NK cells maintain increased levels of glycolysis and OXPHOS by reprogramming cellular metabolic pathways in response to cytokine stimulation. Glucose, the primary fuel for activated NK cells, is metabolized by aerobic glycolysis in the cytosol to pyruvate, which then generates lactate. Unlike other lymphocytes that metabolize pyruvate through the tricarboxylic acid (TCA) cycle, NK cells metabolize pyruvate across the mitochondrial membrane via the Citrate-malate shuttle (CMS) (Fig. 1), which drives the production of OXPHOS and adenosine triphosphate (ATP) [15], a unique metabolic conformation that is regulated by the sterol regulatory element binding protein (SREBP) transcription factor. It controls the expression of two key genes of the CMS: the ATP citrate lyase (ACLY) and the citrate-malate reverse transporter protein SLC25A1. Thus, SREBP activity is crucial for metabolic reprogramming and obtaining higher glycolysis and OXPHOS in NK cells [15]. Furthermore, unlike other types of lymphocytes, NK cells do not use glutamine as a fuel to drive OXPHOS [13].

NK cells express a glucose transporter through which glucose is taken up into the cytoplasm (Glut1). Glucose is metabolized through glycolysis to pyruvate, which is enzyme-catalyzed to lactate and transported outside the cell via the monocarboxylic acid transporter protein (MCT). Some of the pyruvate enters the mitochondria, where most of it is further metabolized via the citrate-malate shuttle (CMS), and only a relatively small amount is metabolized via the tricarboxylic acid cycle (TCA). Pyruvate in the mitochondria is metabolized to Acetyl CoA, which produces NADH. Acetyl CoA binds to oxaloacetate to form citrate, which is then transported out of the mitochondria via the citrate transporter protein (SLC25A1). Citric acid in the cytoplasm is catalyzed by ATP citrate lyase to produce Acetyl CoA and oxaloacetate, which further generates NAD + , which serves as an essential cofactor for the glycolytic enzyme 3-phosphoglyceraldehyde dehydrogenase to maintain glycolysis. Oxaloacetate is converted to malate in the NAD + generating reaction and re-enters the mitochondria via SLC25A1, where it is converted back to oxaloacetate and NADH is produced in the mitochondria. Oxaloacetate can complete the cycle by reacting with another glucose-generating Acetyl CoA to form another citrate. NADH generated by CMS and TCA enters the electron transport chain (ETC), driving OXPHOS and efficient ATP production. In addition, NK cells activated under the stimulation of cytokines (IL15, IL-2, IL-12, IL-18) exhibit a significant increase in glycolysis rate, OXPHOS rate, mitochondrial mass, and metabolic flux to provide the energy support required for NK cell growth and effector molecule synthesis.

### Metabolic characteristics of different subpopulations of NK cells

Human blood NK cells are divided into two main subpopulations: the CD56bright and CD56dim NK cells. CD56dim cells have higher cytotoxicity compared to CD56bright cells, whereas CD56bright NK cells are the main producers of cytokines, including IFN-γ and tumor necrosis factor-α (TNF-α) [1, 16]. The two have different metabolic profiles, with CD56bright cells being more metabolically active than CD56dim cells upon IL-2 or IL-12 stimulation. They preferentially upregulate nutrient receptors and show higher rates of glucose uptake. CD56bright cells require elevated levels of OXPHOS to support cytotoxicity and IFN-γ production in all NK cells [11]. In addition, human NK cells from blood and tissue-resident (e.g., spleen and liver) exhibit different metabolic profiles, and although tissue-resident NK cells also increase trophic receptor expression following stimulation, this increase is less than that of blood NK cells [17]. Human decidual NK cells are a unique type of tissue-resident NK cells. Integration of metabolomics and proteomics data revealed that 29 metabolites involved in the metabolism of glycerophospholipids and glutathione were significantly reduced in decidual NK cells compared to peripheral blood NK cells. In addition, decidual NK cells have an altered redox balance and tend to have more ROS, which may be associated with their reduced cytotoxicity [18].

### NK cell metabolism in pathological states

Evidence suggests that NK cell metabolism is altered in chronic diseases such as cancer, obesity, and viral infections, which are key contributors to NK cell dysfunction. In obesity, NK cells take up lipids from the environment, which interferes with their cellular bioenergetics, leading to metabolic paralysis and a greatly reduced metabolic rate. This metabolic dysfunction is associated with peroxisome proliferator-activated receptor (PPAR)-driven lipid accumulation in NK cells, and PPARα/δ target genes are highly up-regulated in obesity, which inhibits mTOR-mediated glycolysis as well as downstream transcription of cytotoxic particles and IFN-γ production. In addition, obesity severely impedes the ability of NK cells to direct cleaved particles to tumor cells and degranulate them at the synapse, which is critical for NK cells to exert cytotoxicity and thus effectively kill tumors [19]. Glucose metabolism is vital for NK cell-mediated control of Mouse Cytomegalovirus (MCMV) infection, and when aerobic glycolysis is inhibited by cell-specific deletion of Lactate Dehydrogenase A (LDHA), the NK cell's ability to generate a robust effector and memory response to MCMV infection is dramatically diminished [20, 21].

## How TME affects metabolic reprogramming of NK cells

**Fig. 2:**
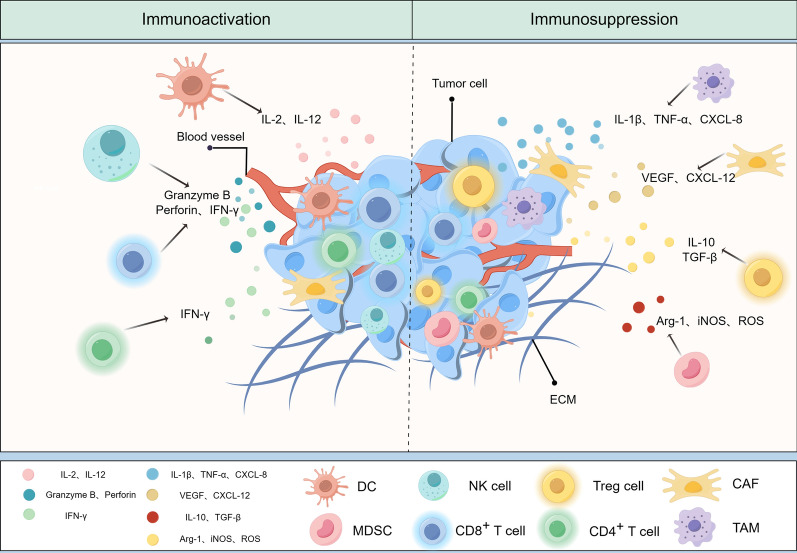
Tumor Microenvironment (drawed by Figdraw). The tumor microenvironment (TME) is the surrounding microenvironment in which tumor cells exist, including the surrounding vasculature, extracellular matrix (ECM), fibroblasts, myeloid-derived inflammatory cells, immune cells, and a variety of cytokines and signaling molecules. Cytokine signaling molecules released by immune cells infiltrated in the TME are involved in mediating immune-suppressive and/or immune-responsive events. *DC* Dendritic cell, *NK CELL* Natural killer cell, *MDSC* Myeloid-derived suppressor cell, *CAF* Cancer-associated fibroblast, *TAM* Tumor-associated macrophage, *IL* Interleukin, *TNF-α* Tumor necrosis factor-α, *CXCL* Chemokine, *VEGF* Vascular endothelial growth factor, *TGF-β* Transforming growth factor-β, *Arg-1* Arginine-1, *iNOS* Inducible nitric oxide synthase, *ROS* Reactive oxygen species

TME refers to the surrounding microenvironment in which tumor cells exist, including surrounding blood vessels, immune cells, fibroblasts, bone marrow-derived inflammatory cells, various signaling molecules, and extracellular matrix (ECM) (Fig. 2) [22, 23]. The immune function of the TME can be shaped by competition for nutrients in the TME, inhibitory effects of accumulated metabolites, and signals that limit metabolic reorganization [24].

### Hypoxia and its metabolite buildup

#### Hypoxia

In rapidly growing and expanding tumors, the overall TME is characterized by hypoxia due to the increase in oxygen consumption and diffusion caused by proliferation and the incomplete internal vascular system of the tumor tissues, resulting in an insufficient supply of oxygen within the tumor tissues. In advanced cancer patients, hypoxic stress shapes NK cells into a tumor-resistant and immunosuppressive phenotype. A way for cells to adapt to hypoxia is to upregulate the protein expression of hypoxia-inducible factor 1α (HIF-1α). Under normoxia conditions, HIF-1α is thought to be dispensable for glycolysis and NK cell metabolic activation, as HIF1α-deficient mouse NK cells have normal levels of glycolysis and OXPHOS responses to IL2 + IL12 cytokine stimulation [13]. However, under hypoxic conditions, HIF-1α appears to be a central player in the activation of NK cell glycolysis. Stable expression of HIF-1α during hypoxia promotes the metabolic shift from OXPHOS to glycolysis in NK cells, overcoming hypoxia-mediated apoptosis and tumor cytotoxicity damage in NK cells maximizing NK cell effector function [25–27]. Notably, researchers have emphasized the importance of pre-cytokine such as IL-2 stimulation on the stable expression of HIF-1α in NK cells, which seems to provide new evidence to achieve high-functioning NK cells in hypoxic microenvironments for adoptive cell therapy [26, 27]. Furthermore, the HIF-1α metabolic pathway in resting NK cells differs from that in activated NK cells, where HIF-1α is a regulator of tryptophan metabolism and cellular nicotinamide adenine dinucleotide (NAD) levels, which prevents the generation of ROS during OXPHOS, thus blocking DNA damage and apoptosis of NK cells under homeostatic conditions [25]. Nevertheless, the question of whether HIF-1α promotes or impairs NK cell effector functions remains a controversial issue. Available evidence suggests that the absence of HIF-1α in NK cells is also beneficial, although this results in attenuated cytotoxicity while showing increased bioavailability of the angiogenic cytokine vascular endothelial growth factor (VEGF), which stimulates non-productive angiogenesis and thus inhibits tumor growth [28]. In addition, Ni et al. found that inhibition of HIF-1α released the antitumor activity of NK cells by establishing single-cell RNA sequencing in mice with conditional targeting deficiency of HIF-1α in NK cells, as evidenced by the inhibition of tumor growth, activation of markers, elevation of expression of the effector molecule IFN-γ, and enrichment of the NF-κB pathway in tumor-infiltrating NK cells [29]. HIF-1α regulation is complex, and the effects of hypoxia and HIF-1α may depend on microenvironmental conditions, which require further study.

#### Adenosine accumulation

Moreover, extracellular adenosine (eADO) buildup is brought on by hypoxia. Cancer cells release large amounts of ATP in hypoxic environments within tumors. These ATP molecules are then converted to adenosine (ADO) by the exonucleotidase enzymes CD39 and CD73, and ultimately the ADO is expelled externally [30, 31]. It has been suggested that the current purinergic signaling effect on the immune response is a balance between the pro-inflammatory, immunosurveillance effects of extracellular adenosine triphosphate (eATP) and the anti-inflammatory, immunosuppressive effects of eADO [32]. Exonucleotidase enzymes are more abundant in solid tumors than in non-malignant tissues, which promotes the hydrolysis of eATP to eADO [30]. G protein-coupled receptors A1, A2A, A2B, and A3 bind to eAOD to initiate downstream signaling pathways. A2A and A2b receptors bind to eAOD to inhibit immune cell activation by producing intracellular cyclic adenosine monophosphate (cAMP) and activating protein kinase A (PKA) during the subacute inflammatory phase that follows tissue damage [31]. The ability of IL-2/nkp46-activated NK cells to produce IFN-γ, tumor necrosis factor α (TNF-α), and macrophage colony-stimulating factor is dramatically inhibited by A2A-CAMP-PKA signaling, which in turn influences cytotoxicity [33]. Furthermore, it appears that ADO signaling has a multipathway effect on the ability of NK cells to kill. It has been discovered that ADO and its analogs hinder the cleavage and lysis of IL-2-activated NK cells by interfering with the killing pathways mediated by FAS ligand and perforin, which are accompanied by increased levels of cAMP [34]. Increased ATP hydrolysis was seen as a result of dysregulated CD39 and CD73 cell surface expression on non-malignant T and NK cell populations in an in vivo study of sezary syndrome. The effective killing of ADCC by NK cells and the restoration of non-malignant CD4 + and CD8 + T cell proliferation can both be achieved through inhibition of the CD39/CD73/ADO pathway [35]. Interestingly, NK cells show the highest amounts of A2A receptor expression, even though A2A receptors are found on the majority of immune cells [36]. Research has revealed that the A2A receptor functions as a checkpoint to restrict NK cell development by directly impeding NK cell maturation in the TME. The percentage of terminally developed NK cells in the TME can rise when the A2A receptor is conditionally deleted [37]. Thus, NK cell effector function may be restored and immune surveillance may be improved by targeting ADO-related signaling molecules in the purinergic signaling cascade.

### Acidic TME shaped by glucose restriction and its metabolic end products

#### Glucose limitation

Most tumors are characterized by a high degree of glycolysis, even in the presence of oxygen, and cancer cells are thought to rely more on glycolysis than mitochondrial OXPHOS for energy production, i.e., aerobic glycolysis or the "Warburg effect". High glucose consumption in cancer cells leads to low levels of glucose available for TME and a lack of fuel for NK cell activation resulting in metabolic limitation, which affects NK cell activation, impairs IFN-γ production, and blunts the NK cell antitumor response [11, 38]. However, whether extrinsic cellular competition for nutrients or intrinsic cellular reprogramming leads to metabolic dysregulation of tumor-promoting immune cells remains a controversial issue. The study by Bradley et al. challenged the hypothesis that cancer cells induce immune cell nutrient deficiencies and dysfunction in the TME by competing for glucose with the TME. Their findings suggest that glucose is preferentially utilized by tumor-associated immune cells, in contrast to cancer cells that exhibit the greatest uptake of glutamine. Different cells in the TME have different nutrient uptake programs, which are cell-intrinsically programmed through mTORC1 signaling as well as glucose- and glutamine-related gene expression. This interfered with glutamine metabolism in cancer cells, which increases glucose uptake to generate energy using glutamine metabolism as a specific strategy to impede cancer cell growth while increasing glucose consumption and consequently altering the immune phenotype in TME [39].

#### LDH-derived lactic acid accumulation

Lactate dehydrogenase (LDH) is a key enzyme in glycolysis, and it catalyzes the reversible conversion of pyruvate to lactic acid and NADH to NAD, the last step in the glycolytic process [40] (Fig. 1). High serum LDH levels are frequently linked to a bad prognosis in a variety of cancer types, such as melanoma, bladder, nasopharyngeal, pancreatic, non-small-cell lung carcinoma, etc. [41–45]. Elevated serum LDH levels were linked to a lower chance of survival for solid tumors, specifically prostate cancer, renal cell carcinoma, and melanoma, according to a meta-analysis of 76 studies [46]. The potential of variations in serum LDH levels as biomarkers to forecast treatment outcomes and dynamically monitor therapy response has also been emphasized by several clinical investigations. In metastatic colorectal cancer, for instance, serum LDH can function as a measure of tumor angiogenesis activation, hence predicting the effectiveness of bevacizumab in suppressing angiogenesis [47]. LDH seems a biomarker for the anti-PD-1 immunotherapy result in esophageal squamous cell cancer [48]. Overexpression of LDH in tissues supports a range of malignant biological behaviors of tumor cells, including the epithelial-to-mesenchymal transition (EMT) [49], promotion of cell invasion and migration [50–52], angiogenesis [53], and cytoskeletal remodeling [54]. These findings are supported by numerous in vitro and in vivo experiments. It is also associated with resistance to anticancer therapies, and it has been found that impeding glycolytic metabolism in cancer cells through LDH inhibition can overcome resistance to chemotherapeutic agents [55–60]. Notably, serum LDH levels do not always correspond with the level of LDH in tumor tissues, and it is unclear if elevated serum LDH levels are a result of tumor cell leakage or the rapid growth of metastases destroying nonmalignant tissues [61]. Still, tissue LDH levels and serum LDH levels can cooperate and complement one another.

One of the features of TME is the high enrichment of lactate, and LDHA has a considerable ability to affect immunity in TME by boosting lactate production. On the one hand, tumor-associated fibroblasts, the primary stromal cells in TME, use lactate produced by LDH as fuel. LDH facilitates the mutual nutrition exchange between stromal and tumor cells and encourages the growth of tumors [62]. Lactate, on the other hand, makes the TME acidic. Acidic TME inhibits the antitumor immune response, supports Treg cell differentiation and tumor-associated macrophage polarization toward M2, encourages the recruitment of myeloid suppressor cells, and prevents dendritic cell maturation, limits NK cell toxicity, which suppresses intrinsic and acquired immunity [61]. NK cells are affected by high TME lactate levels as follows: Lactate is a metabolic end product of glycolysis, and its high enrichment is one of the characteristics of the TME, which is increasingly being studied in closely related to the immunosuppressive microenvironment. Lactate causes acidification of TME, and lactate uptake by NK cells leads to intracellular acidification and impaired energy metabolism. 15 mM lactate has completely blocked IFN-γ production, and lactate concentrations higher than 20 mM led to apoptosis of T cells and NK cells [63]. Similar results were obtained in liver-resident NK cells treated with lactic acid [64]. Ge et al. verified in vivo and in vitro experiments in a pancreatic cancer model that the SIX1/LDHA axis promotes the accumulation of tumor lactate and thus inhibits the function of NK cells [65]. In addition, lactate-induced acidification inhibits the nuclear factor of activated T-cells (NFAT) for transcription, an important transcription factor involved in the transcriptional control of IFN-γ, which likewise leads to a decrease in IFN-γ production by NK cells [63]. Elevated lactate levels not only directly limit the cellular function of NK cells, but also indirectly inhibit NK cells by increasing the number of MDSCs.

### Decreased available amino acids

#### Arginine

Tumor cells consume large amounts of amino acids, which synergize with tumor-associated cells to create a nutritionally depleted and immunosuppressive TME. Additionally, elevated catabolism of tryptophan and arginine (Arg) is a common hallmark of TME [66]. Previous data suggest that Arg plays a critical role in T cell activation and proliferation, and is also required for optimal proliferation of NK cells [67, 68]. Arg depletion reduces NK cell proliferation and IFN-γ production while decreasing NK ζ-chain expression inhibits activation signaling to control NK cell cytotoxicity [69]. A recent study indicated that mitochondria-derived damage-associated molecular pattern (mitoDAMP) inhibited NK cell-mediated cytotoxicity, IFN-γ production, T cell proliferation, and in vivo activation of antiviral T cells. Mass spectrometry analysis of mitoDAMP revealed enrichment of Arg and its enzymatic activity products, and further addition of Arg or arginase inhibitors could reverse the inhibitory effect of MitoDAMP preparations. Further supporting that Arg depletion is responsible for the altered immune polarity [70]. However, it has also been reported that NK cell granule exocytosis and cytotoxicity are not related to extracellular Arg [71]. Although the effects of Arg deprivation on NK cytotoxicity are controversial, they all point to the fact that lack of Arg impairs IFN-γ expression in NK cells through a post-transcriptional mechanism [69–71].

#### Tryptophan

Many cancer and tumor-associated cells in TME [e.g., tumor-associated macrophages (TAMs), tumor-associated dendritic cells (DCs), and fibroblasts] express increased indoleamine 2,3-dioxygenase (IDO) and tryptophan-2,3-dioxygenase (TDO), and large quantities of tryptophan are converted to kynurenine by the enzymes [72, 73]. On the one hand, this leads to tryptophan depletion and reduced availability of essential amino acids, inhibiting tumor-infiltrating lymphoid (TIL) cell responses. On the other hand, accumulation of kynurenine in TME enters TILs via the amino acid transporter protein SLC7A5 and impairs TIL proliferation and effector function [73]. In NK cells, exposure to IDO-derived kynurenine also induces apoptosis via reactive oxygen species-mediated pathways and also decreases the expression of the NK cell activation receptor NKG2D and natural cytotoxicity triggering receptor 1 (NCR1, also known as NKp46). But the limiting effect of kynurenine is selective and it does not affect the cytotoxicity effects mediated by NKp30 or CD16 [74, 75]. Inhibition of IDO may be a potential anticancer target. Silencing of IDO in ovarian cancer cells enhances cancer cell sensitivity to NK cells in vitro, and in vivo and manifests as inhibition of tumor growth, reduction of peritoneal dissemination, and promotion of NK cell accumulation in the tumor stroma [76].

#### Glutamine

Previously, we mentioned that cancer cells consume the highest amount of glutamine, while immune cells consume the highest amount of glucose [39]. The predatory uptake of glutamine by tumor cells in the TME results in limited utilization of glutamine by immune cells. In NK cells, SLC7A5-mediated glutamine uptake is required to regulate c-MYC-dependent NK cell activation. Glutamine-deficient NK cells exhibit reduced c-MYC protein expression, growth restriction, and impaired immune function. However, the inhibition of glutamine catabolism did not affect NK cells driving OXPHOS, suggesting that although glutamine is not an essential fuel for NK cells, glutamine is important for maintaining the signaling of metabolic regulators such as MYC [13, 77]. Targeting glutamine metabolism enhances NK cell-based therapies by impairing tumor fuel supply without decreasing NK cell function.

## Strategies for targeting immunometabolism to optimize NK cell immunotherapy

### Targeted hypoxia and its metabolites

#### Targeted hypoxia

Although it is not yet clear whether the expression of HIF-1α in NK cells has a promoting or impairing effect on NK cell effector function, downregulation of HIF-1a signaling within the tumor itself can also enhance immune activity. A variety of HIF-1α inhibitors are being developed to improve cancer immunotherapy. For adoptive cellular therapy, preactivated NK cells using IL-2 can restore the killing potential of NK cells exposed to hypoxic TME. When NK cells are in an active proliferative state, the proliferative signaling of NK cells is sufficient to amplify hypoxia-induced activation of ERK1/2 and STAT3, thereby shifting to anti-apoptotic and pro-survival pathways to resist the deleterious hypoxic immunosuppressive environment [27]. In addition, dysfunctional vasculature in tumor cells contributes to hypoxia and tumor drug resistance, thus normalizing the vasculature to reduce hypoxia in the TME takes on a strong role. Treatment with a novel Fc-VEGF chimeric antibody drug (Fc-VFD) inhibits the secretion of pro-angiogenic factors VEGF-A and IL-6 by cancer cells in TME, suppressing excessive angiogenesis and overcoming hypoxia resistance in cancer cells. Single-cell RNA sequencing also revealed that it inhibited M2 macrophage polarization and increased immune cell infiltration, including cytotoxic T cells, NK cells, and M1 macrophages [78]. The use of the anti-vascular drug 5,6-Dimethylxanthenone-4-acetic Acid (DMXAA) in combination with the HIF-1α inhibitor Digoxin inhibited tumor growth and stimulated immunity in a melanoma model, demonstrating a significant increase in the percentage of CD8 cytotoxic lymphocytes and NK cells [79]. In addition, HIF-1α is associated with PD-L1, and HIF-1α binds to hypoxia-responsive elements in the PD-L1 promoter to regulate PD-L1 expression. Combination therapy with a PD-L1 antibody and a HIF-1α inhibitor may improve the immune response of patients and enhance immune efficacy [80]. Castration-resistant prostate cancer (CRPC) cells highly express PD-L1, and the cytotoxicity of NK cells against hypoxia-induced CRPC cells was enhanced by the inhibition of JAK1, STAT3 (upstream regulators of PD-L1) in combination with PD-L1 antibody [81].

#### Targeting adenosine

ADO has a key role in remodeling the immunosuppressive TME. The utilization of immunotherapy or inhibitors of signaling to promote immune cells against malignant tumors may be a promising therapeutic approach. Recently, a human anti-CD73 monoclonal antibody Oleclumab has completed phase I clinical trials and demonstrated a manageable safety profile when administered alone or in combination with Durvalumab and showed antitumor activity in immunotherapy-resistant tumor types [82, 83]. ARL67156, a dual inhibitor of CD39/73, enhanced the cytotoxic effects produced by NK tumor cell lines [84]. In addition, it was found that antagonism of A2A ADO receptors decreased the percentage of CD56bright NK cells and promoted the accumulation of highly cytotoxic CD56dim NK cells. This suggests that A2A receptor antagonism enhances adoptive NK cell immunotherapy [37]. There have been several reports using combinations of A2A receptor antagonists and NK cell-based therapies that can promote NK cell-mediated antitumor immunity [85–88].

### Targeted glucose metabolism and its metabolic end products

#### Targeted glucose metabolism

In the TME where tumors consume glucose and have high rates of glycolysis, inhibition of glycolysis may be a promising therapeutic strategy to alleviate glucose deprivation and limit tumor growth in TME. Single or combination therapies with the glycolysis inhibitor 2-deoxyglucose (2-DG) have entered clinical trials [89, 90]. However, glucose is also a key fuel for the activation of NK cells, and one has to question whether targeting the glycolytic pathway would inhibit glycolysis in NK cells in the context of TME thereby impairing the ability of NK cells to kill tumor cells. The effect of 2-DG on NK cell cytotoxicity is currently unknown. One data showed decreased NK cell proliferation and cytotoxicity in CMV-infected mice treated with 2-DG [21]. In contrast, other data suggests that it is glucose starvation rather than 2-DG treatment that impairs NK cell cytotoxicity [91]. However, the preactivation of NK cells with cytokines for adoptive cell therapy may be able to overcome metabolic inhibition. It has been shown that even in the presence of 2-DG, NK cells preactivated with IL-12/15/18 exhibited higher cytotoxic activity than control NK cells. This suggests that cytokine-induced memory-like (CIML) NK cells can maintain higher anti-tumor activity under glycolysis-restricted conditions (e.g., TME) [92]. In addition to this, some inhibitors were used to assess the effect on glycolysis in NK cells. Administration of the aldose reductase inhibitor Fidarestat downregulated AKR1B10 expression in NK cells and promoted NK cell glycolysis to enhance its killing activity against hepatocellular carcinoma cells [93]. Adoptive transfer of NK cells treated with MB05032, an inhibitor of the gluconeogenic enzyme FBP1, restored NK cell glycolysis and effector functions and slowed down tumor growth in a murine lung cancer model [94].

#### Targeting lactate

Targeting lactate metabolism to regulate the pH of the TME is a promising approach due to the damage to NK cells caused by lactate and lactate-induced acidification of the TME. This includes both aspects of targeting lactate anabolism and targeting lactate transport. One key to the strategy of targeting lactate anabolism is LDH-A, a key enzyme that catalyzes the conversion of pyruvate to lactate during glycolysis. Several small molecule inhibitors targeting LDH-A have been applied in preclinical trials and shown to be effective in inhibiting tumor growth. These include substrate (pyruvate)-competitive inhibitors: oxalate [95]; cofactor (NADH)-competitive inhibitors: cotton phenol, quinoline 3-sulfonamides [96–98]; and dual competitive inhibitors (substrate and cofactor): N-hydroxy-indole (NHI) [99, 100]. Some of these molecules are in clinical trials, but no LDH-A inhibitor has yet been approved for clinical use, and the clinical utility of LDHA inhibitors may be limited by their non-selective toxicity or complex interactions with other cellular components. Second, targeting lactate transport. Cancer cells take advantage of the widespread expression of two monocarboxylic acid transporter (MCT) protein isoforms, namely, MCT1, which dominates lactate import, and MCT4, which dominates lactate export, to establish a lactate shuttle in cancer cells. The use of MCT inhibitors to block the release of lactate from cancer cells reduces TME acidification and causes lactate accumulation in cancer cells to reach toxic concentrations in tumor cells, reducing their growth and inducing apoptosis. An MCT1 inhibitor, AZD3965, is in clinical trials and modulates tumor immune cell infiltration, specifically increasing NK cell and DC cell abundance and maturation within the tumor [101, 102]. In addition, monotherapy using systemic bicarbonate buffering that neutralizes tumor acidity enhances IFN-γ expression in NK cells and increases the number of NK cells in tumor-growing lymphoid organs, and delays tumor growth in an NK cell-dependent manner [103]. However, the results of three phase I/IIa clinical trials of oral sodium bicarbonate tolerance (NCT01846429, NCT01198821, NCT01350583) suggest that it is not easy to implement mono buffered therapies in the clinic. The trials were unsustainable because patients were experiencing diminished taste and strong gastrointestinal reactions thus leading to poor compliance [104].

### Targeted amino acid metabolism

#### Targeting arginine

Low Arg levels affect NK cell proliferation. The small molecule arginase inhibitor CB-1158 blocks Arg depletion by myelomonocytic arginase expression in the TME, activates NK cells, promotes their infiltration into the TME, and acts synergistically with checkpoint inhibition to promote tumor clearance [67]. Furthermore, in a study that included 18 colorectal cancer patients who underwent tumor resection, preoperative Arg supplementation was found to lead to an increase in tumor-infiltrating CD16 and CD56 NK cells by histopathological analysis of biopsies [105].

#### Targeting tryptophan

Based on its effects on NK cells as well as other immune cells, IDO inhibition has emerged as a potential target for anticancer therapies. Most inhibitors bind to IDO enzymes and prevent the conversion of tryptophan to kynurenine, resulting in toxic effects on NK cells and other immune substrates. The use of IDO/TDO-IN-2, a dual inhibitor targeting IDO3 and TDO1, reversed the inhibition of NK cell-mediated antibody-dependent cytotoxicity (ADCC) by oncolytic-positive cancer-associated fibroblasts and helped to attenuate Trastuzumab resistance in HER2 positive breast cancer [106]. In a study on sarcoma, an IDO inhibitor (GDC-0919) was in use. Although GDC-0919 alone or in combination with anti-PDL1 neither show antitumor activity nor affect tumor immune cell infiltration, GDC-0919 was found to induce downregulation of granzyme expression, as well as upregulation of inhibin subunit beta A (inhba) and E3 Ubiquitin ligase Dtx4 [107]. It has been reported that inhba plays a crucial role in inhibiting NK cell proliferation and granzyme B production, leading to impaired tumor susceptibility to NK cell-mediated killing [108]. These results indicate a potential role for the IDO pathway in controlling NK function [107]. By delivering immunogenic cell death (ICD) stimuli combined with interference with the immunometabolism effects of the IDO-1 pathway, a dual-delivery liposomal carrier with mitoxantrone and cholesterol fingolimod as prodrugs was developed, and the data showed that liposomal delivery was very effective for inducing a chemoimmunotherapeutic response with the involvement of NK, which has been successfully validated for chemoimmunotherapy in mouse models of breast and renal cancer [109]. In addition, multi-combination immunotherapy, i.e., nanoscale reduced graphene oxide-mediated photothermal therapy synergized with IDO inhibition and PD-L1 blockade, designed by Yan et al. was shown to directly kill tumor cells and induce synergistic anti-tumor immunity. Experiments in vivo further demonstrated that the immune response enhanced the production of tumor-infiltrating lymphocytes, including CD4 T cells, CD8 T cells, and NK cells, as well as INF-γ [110].

#### Targeted glutamine

As mentioned previously, we mentioned that targeting glutamine metabolism could enhance NK cell-based therapies by impairing tumor fuel supply without decreasing NK cell function. Based on this idea JHU083 was used in preclinical studies. JHU083 is a prodrug of 6-diazo-5-oxo-L-norleucine (DON), a glutamine antagonist that can interfere with tumor cell metabolism by inhibiting glutaminase and a variety of enzymes in tumors that require glutamine thereby increasing the availability of glucose and oxygen in the TME and reducing TME acidification [111]. These alterations are beneficial in supporting NK cell effector function. We have already known the importance of SLC7A5-dependent amino acid uptake in NK cells for the stable and sustained expression of c-MYC [13]. This has led researchers to focus on therapeutic strategies that target NK cells to upregulate amino acid transporter proteins to enhance metabolic adaptations in NK cells: ① Chimeric Antigen Receptor (CAR)-expressing NK cells armed with overexpressed transporter proteins; ② Overexpression of transporter protein positive regulators (e.g., c-MYC, YBX3) and knockdown/silencing of transporter protein negative regulators (e.g., MARCH1); ③ Enhance the expression of amino acid transporter proteins specifically using cytokines such as IL-15, IL-18 [72]. In addition, MYC in NK cells is degraded by glycogen synthase kinase 3 (GSK3), and several clinical trials (NCT01287520) targeting GSK3 inhibitors (NCT01214603) are ongoing [112, 113]. Notably, SREBP activity is not only critical for metabolic reprogramming and obtaining higher glycolysis and OXPHOS in NK cells, but is also required for c-MYC protein expression [15, 114]. Activation of SREBP transcription factors can be effectively inhibited by cholesterol and oxysterols, and fluoxetine, and betulinic acid alcohols have been shown to suppress SREBP activation and have shown promising antitumor effects in preclinical studies [115, 116].

## Conclusion and outlook

Numerous NK cell-based immunotherapies, including ex vivo NK cell activation, NK cell overlay therapy, and CAR-NK cell therapy, are quickly developing in the context of molecularly targeted treatments. Among them, CAR-NK cell therapy is regarded as a milestone breakthrough in anti-tumor immunotherapy is CAR-NK cell treatment. Because of the special biological characteristics of NK cells, CAR-NK treatment has the distinct benefits of low toxicology and multi-cell origin. Even the use of allogeneic NK cells prevents allogeneic responses and lowers the risk of neurotoxicity and serious toxic side effects including acute cytokine release syndrome (CRS) and graft-versus-host disease (GVHD). Nevertheless, there are still several obstacles in the way of its clinical application in solid tumors, including those related to the creation of CAR-NK cells, their ability to migrate to the tumor site, and their survival and durability in immunosuppressed TME. In particular, the importance of "adaptive" regulation of CAR-NK cells in TME suggests that the eventual development of NK cell exhaustion remains a major obstacle to the success of NK cell immunotherapy treatments, which are closely related to TME. Understanding the interactions in the metabolic biology of NK cells and specific TMEs will help to identify the necessary NK cell modifications and the relevant choices of NK cell sources to optimize NK cell-based immunotherapies as a cancer immunotherapy boon.

In addition, immunometabolism is a fascinating area of research that may be key to successful cancer immunotherapy. However, targeting immune metabolism during immunotherapy is challenging. Because tumor cells and immune cells share similar metabolic pathways for energy acquisition, targeting tumor cell metabolism may impair immune cell metabolism in the context of TME and thus diminish their effector function. An example is the uncertainty of the glycolysis inhibitor 2-DG, which targets glucose metabolism, on NK cell metabolism. Future studies should deepen the differences between the metabolic mechanisms of cancer cells and immune cells in the context of TME and adjust the strategy of targeting metabolic regulation.

## Data Availability

Not applicable.

## Abbreviations

NK: Natural killer
TME: Tumor microenvironment
OXPHOS: Oxidative phosphorylation
IFN-γ: Interferon γ
TCA: Tricarboxylic acid
CMS: Citrate-malate shuttle
SREBP: Sterol regulatory element binding protein
ATP: Adenosine triphosphate
ACLY: ATP citrate lyase
PPAR: Peroxisome proliferator-activated receptor
MCMV: Mouse cytomegalovirus
LDHA: Lactate dehydrogenase A
ECM: Extracellular matrix
HIF-1α: Hypoxia-inducible factor 1α
NAD: Nicotinamide adenine dinucleotide
VEGF: Vascular endothelial growth factor
PKA: Protein kinase A
NFAT: Uclear factor of activated T-cells
Arg: Arginine
mitoDAMP: Mitochondria-derived damage-associated molecular pattern
IDO: Indoleamine 2,3-dioxygenase
TDO: Tryptophan-2,3-dioxygenase
TIL: Tumor-infiltrating lymphoid
NCR1: Natural cytotoxicity triggering receptor 1
CRPC: Castration-resistant prostate cancer
2-DG: 2-Deoxyglucose
CIML: Cytokine-induced memory-like
NHI: N-hydroxy-indole
MCT: Monocarboxylic acid transporter
ADCC: Antibody-dependent cytotoxicity
ICD: Immunogenic cell death
CAR: Chimeric antigen receptor
GSK3: Glycogen synthase kinase 3
DMXAA: 5,6-Dimethylxanthenone-4-acetic acid
Inhba: Inhibin subunit beta a
TNF-α: Tumor necrosis factor-α
ADO: Adenosine
eATP: Extracellular adenosine triphosphate
eADO: Extracellular adenosine
cAMP: Cyclic adenosine monophosphate
LDH: Lactate dehydrogenase
EMT: Epithelial-to-mesenchymal transition
NFAT: Nuclear factor of activated T-cells
CRS: Cytokine release syndrome
GVHD: Graft-versus-host disease
